# Flow Cytometric Measurement of Blood Cells with BCR-ABL1 Fusion Protein in Chronic Myeloid Leukemia

**DOI:** 10.1038/s41598-017-00755-y

**Published:** 2017-04-04

**Authors:** Liza Löf, Linda Arngården, Ulla Olsson-Strömberg, Benjamin Siart, Mattias Jansson, Joakim S. Dahlin, Ingrid Thörn, Lisa Christiansson, Monica Hermansson, Anders Larsson, Erik Ahlstrand, Göran Wålinder, Ola Söderberg, Richard Rosenquist, Ulf Landegren, Masood Kamali-Moghaddam

**Affiliations:** 1 Dept. of Immunology, Genetics & Pathology, Science for Life Laboratory, Uppsala University, Uppsala, Sweden; 2 Dept. of Medical Science and Division of Hematology, University Hospital, Uppsala, Sweden; 3 Department of Anthropology, University of Vienna, Vienna, Austria; 4 Dept. of Medicine, Karolinska Institutet, Karolinska University Hospital, Stockholm, Sweden; 5 Dept. of Medical Science and Division of Biochemical Structure and Function, University Hospital, Uppsala, Sweden; 6 Dept. of Medicine, Division of Hematology, Örebro University Hospital, Örebro, Sweden; 7 Dept. of Pharmaceutical Biosciences, Uppsala University, Uppsala, Sweden

## Abstract

Chronic myeloid leukemia (CML) is characterized in the majority of cases by a t(9;22)(q34;q11) translocation, also called the Philadelphia chromosome, giving rise to the BCR-ABL1 fusion protein. Current treatment with tyrosine kinase inhibitors is directed against the constitutively active ABL1 domain of the fusion protein, and minimal residual disease (MRD) after therapy is monitored by real-time quantitative PCR (RQ-PCR) of the fusion transcript. Here, we describe a novel approach to detect and enumerate cells positive for the BCR-ABL1 fusion protein by combining the *in situ* proximity ligation assay with flow cytometry as readout (PLA-flow). By targeting of the BCR and ABL1 parts of the fusion protein with one antibody each, and creating strong fluorescent signals through rolling circle amplification, PLA-flow allowed sensitive detection of cells positive for the BCR-ABL1 fusion at frequencies as low as one in 10,000. Importantly, the flow cytometric results correlated strongly to those of RQ-PCR, both in diagnostic testing and for MRD measurements over time. In summary, we believe this flow cytometry-based method can serve as an attractive approach for routine measurement of cells harboring BCR-ABL1 fusions, also allowing simultaneously assessment of other cell surface markers as well as sensitive longitudinal follow-up.

## Introduction

Chronic myeloid leukemia (CML) originates from a pluripotent hematopoietic stem cell (HSC) that acquires the t(9;22)(q34;q11) translocation, i.e. the hallmark cytogenetic aberration of CML. This translocation, commonly referred to as the Philadelphia (Ph^1^) chromosome, leads to a fusion protein, where the 5′ part of the *BCR* gene is fused to the 3′ part of the *ABL1* gene^1–4^, results in significantly increased tyrosine kinase activity and cell proliferation. While more than 95% of CML patients carry the *BCR-ABL1* fusion gene^5^, a minor proportion of acute lymphocytic leukemias (ALL) and acute myeloid leukemias (AML) may also harbor the *BCR-ABL1* fusion^6^. The characteristic t(9;22) translocation is detected by routine cytogenetics through karyotyping or fluorescence *in situ* hybridization (FISH)^7^, or the *BCR-ABL1* fusion transcript can be demonstrated by real-time quantitative PCR (RQ-PCR)^8, 9^. Due to various possible breakpoints of the *BCR-ABL1* fusion in different patients, RQ-PCR must encompass the most common variants, i.e. the major variant (210 kDa) and the minor variant (190 kDa)^10^, where the former is predominant in CML^11, 12^.

Today’s successful treatment of CML patients is based on blocking of the ATP-binding site in the ABL1 domain, hence inhibiting its tyrosine kinase activity^13^. Tyrosine kinase inhibitors (TKIs) such as imatinib and nilotinib efficiently inhibit the oncogenic effects of the BCR-ABL1 fusion protein. The current gold standard method for monitoring therapy responses in CML is based on RQ-PCR of the *BCR-ABL1* transcripts to determine whether the patient achieves and remains in molecular remission (<0.1%) or if *BCR-ABL1* positive cells still persist, i.e. minimal residual disease (MRD). Due to the risk of developing resistance mutations in the ABL1 domain, *BCR-ABL1* transcript levels are monitored every third month to get early indications of any increased MRD levels signifying an impending clinical relapse. In recent years, attempts have been made to discontinue therapy in CML patients in longstanding molecular remission^14, 15^. However, even patients with complete molecular responses to TKIs may relapse after discontinuing treatment as a consequence of small numbers of remaining leukemic stem cells (CD34^+^/CD38^−^)^14–17^.

**Figure 1:**
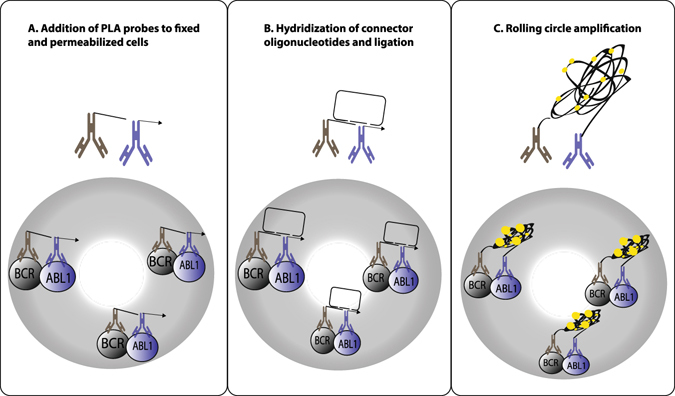
Detection of cells expressing BCR-ABL1 with PLA-flow. The assay employs a pair of oligonucleotide-conjugated antibodies (PLA probes) with affinity for the BCR and ABL1 domains of the fusion protein in fixed and permeabilized cells (**A**). The oligonucleotides on PLA probes remaining in close proximity after washes serve as templates for hybridization of two additional DNA oligonucleotides, guiding their ligation into DNA circles (**B**). After ligation, the DNA circles are locally amplified by rolling circle amplification (RCA) to generate RCA products that are visualized and detected by addition of complementary fluorophore-labeled oligonucleotides (**C**), followed by detection of labeled cells via flow cytometry.

Although RQ-PCR is a powerful method for routine diagnostics used worldwide, the possibility to apply sensitive, flow cytometry to detect cells expressing BCR-ABL1 fusion proteins could prove convenient both at diagnosis and at follow-up. Accordingly, we describe a novel approach to monitor CML patients by quantifying leukocytes harboring the BCR-ABL1 fusion at the protein level. This method, herein called PLA-flow, uses the *in situ* proximity ligation assay (*in situ* PLA)^18, 19^ to detect the BCR-ABL1 fusion protein inside cells (Fig. 1). The PLA-flow protocol uses one antibody directed against the BCR part of the fusion protein and another one against the ABL1 part; each antibody carries a DNA oligonucleotide that, once in proximity, guide the formation of a DNA circle upon hybridization and ligation of two subsequently added DNA oligonucleotides. The ligated DNA circle can then be amplified by rolling circle amplification (RCA) and the localized RCA products are detected by hybridization of fluorophore-coupled oligonucleotides. Finally, the fluorescence intensity of individual cells is measured by flow cytometry. We demonstrate that PLA-flow is a rapid, sensitive and specific method with results that correlate well with those of RQ-PCR for the fusion transcript. In addition, usage of flow cytometry gives the advantage of simultaneously investigation of surface markers commonly applied in the clinical routine setting.

## Results

### Establishing the flow cytometry-based PLA assay

**Figure 2:**
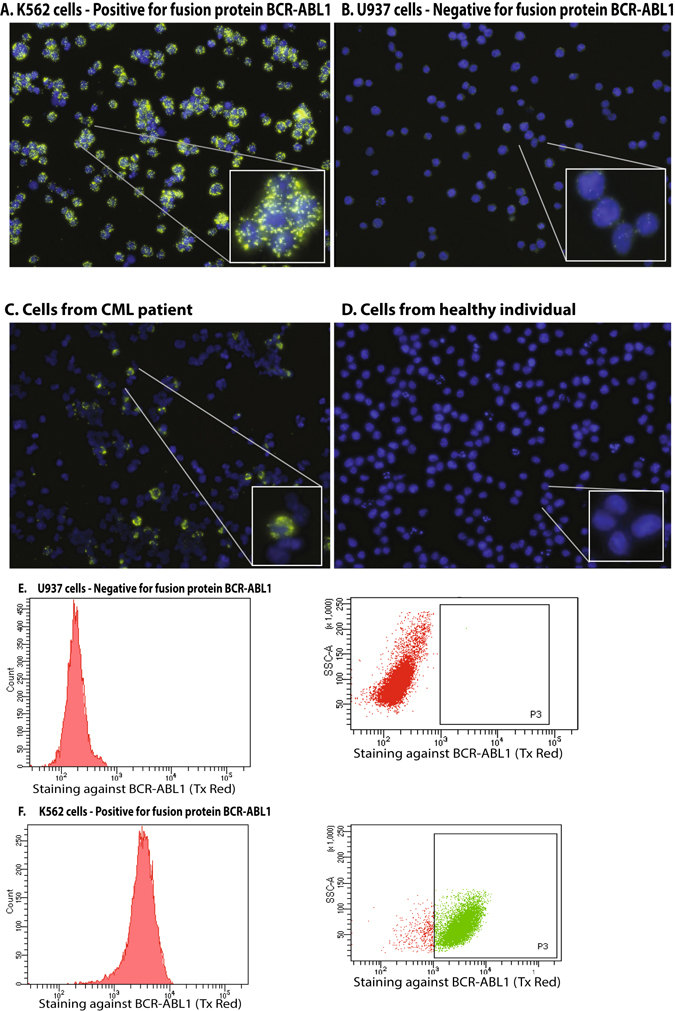
*In situ* PLA detection by fluorescence microscopy of BCR-ABL1 fusion protein. (**A**) Detection of the BCR-ABL1 positive cell line K562, (**B**) Detection of the BCR-ABL1 negative cell line U937, (**C**) cells from a patient treated for CML, (**D**) nucleated blood cells from a healthy individual. Signals were recorded using (**E**) PLA-flow for detection of BCR-ABL1 in the BCR-ABL1 negative cell line U937 compared to (**F**) the BCR-ABL1 positive cell line K562. Experiments were repeated more than three times.

Conditions for detecting BCR-ABL1 fusion proteins through *in situ* PLA with flow cytometry as readout, PLA-flow (Fig. 1), were established using K562 cells that carry the BCR-ABL1 fusion, while the BCR-ABL1 negative cell line U937 was used as a negative control. Briefly, cells to be analyzed were fixed and permeabilized before being subjected to reagents for PLA-flow, followed by detection of labeled cells by microscopy or flow cytometry. The K562 cell line exhibited brightly positive staining in the BCR-ABL1 assay, whereas U937 cells were negative, confirming the selectivity of the assay. Similarly, cells positive for the BCR-ABL1 fusion protein were detected in samples from CML patients, while healthy control samples lacked specifically labeled cells (Fig. 2).

**Figure 3:**
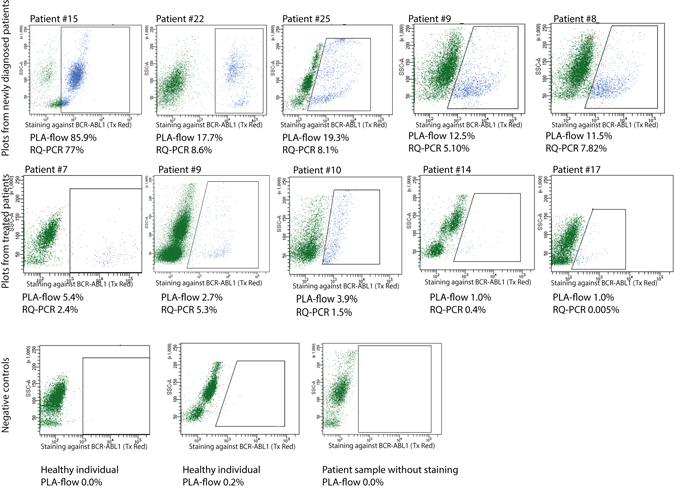
PLA-flow detection of BCR-ABL1 positive cells from patients diagnosed with CML and normal controls. The top row dot-plots are examples from analysis with PLA-flow of newly diagnosed patients, while the middle row represents patients previously treated or under treatment. At the time for sample collection, patients 7 and 9 were treated with imatinib, patient 10 did not receive any treatment and patients 14 and 17 were treated with dasatinib. The cells were gated first for side and forward scatter and then a positive gate was placed around cells that were considered positive compared to a negative control. The negative control used for gating was a patient sample that was not subjected to any PLA probes. The bottom row displays examples of the negative controls, i.e. two samples from healthy individuals and one sample from a patient where no PLA probes were added. The patient samples were also analyzed with RQ-PCR to estimate *BCR-ABL1* transcript levels. Results from both analyses are indicated below each plot where applicable.

Once the staining protocol was established, the PLA-flow assay was applied to analyze nucleated cells in blood samples from five patients with newly diagnosed with CML and five samples from CML patients under treatment. The samples were tested on the same day at the clinical laboratory using the standard RQ-PCR method for *BCR-ABL1* major detection^8^. For each CML sample analyzed, a negative control with blood from an age-matched healthy individual was also analyzed, and as a technical negative control the proximity probes were omitted from CML patient samples. Overall, the PLA-flow gave results similar to those from RQ-PCR, with minor differences observed in some patients (Fig. 3).

In addition, both PB and bone marrow cells from two CML patient samples, one newly diagnosed with a high number of *BCR-ABL1* transcripts and one treated patient with a low number of *BCR-ABL1* transcripts, were analyzed by PLA-flow. The results were comparable with a higher number of positive cells in the PB sample for both patients (Supplemental Table S1).

### Applying PLA-flow for MRD detection

**Figure 4:**
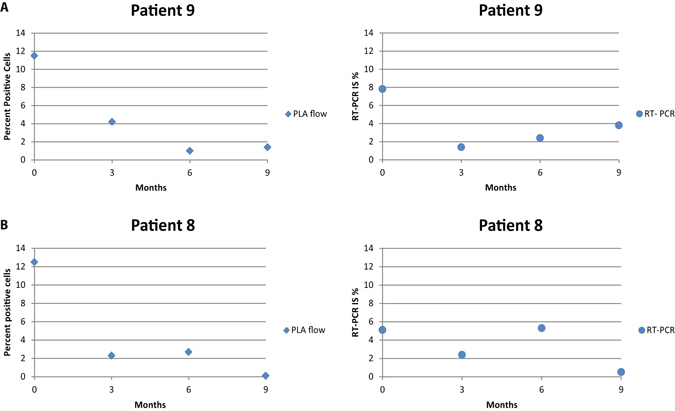
Long-term follow-up of newly diagnosed patients. Two CML patients were followed from the time of initial diagnosis, by measuring BCR-ABL1 positive cells using PLA-flow and *BCR-ABL1* transcript levels using RQ-PCR, every 3 months over a period of 9 months. (**A** and **B**) Display results for patient 1 and 2, respectively, analyzed with PLA-flow (diamonds) and RQ-PCR (circles).

**Figure 5:**
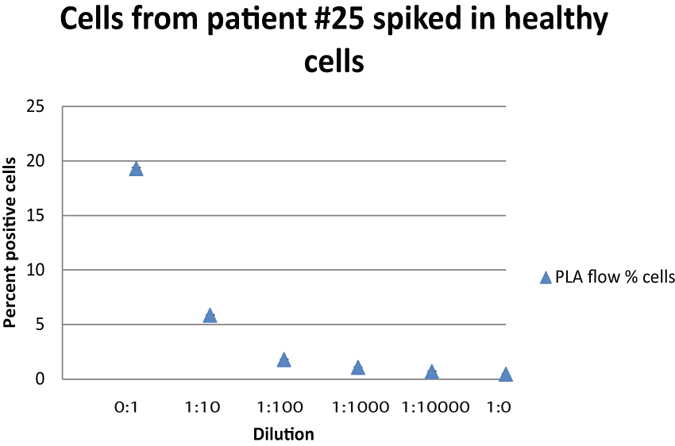
Demonstration of the sensitivity of PLA-flow. Blood from a newly diagnosed CML patient was spiked in blood from a healthy individual in a ten-fold dilution series. Samples were analyzed with PLA-flow to detect positive cells expressing the fusion protein BCR-ABL1. The X-axis indicates the dilutions, while the Y-axis displays percentages of cells expressing BCR-ABL1. Error bars represent standard deviation (SD).

Since monitoring MRD is crucial during treatment of CML patients, we followed two CML patients with PLA-flow and RQ-PCR at the time of diagnosis and at three-month intervals for nine months, and the two methods revealed consistent results (Fig. 4). We next investigated the sensitivity of PLA-flow by spiking blood from a newly diagnosed CML patient with 19.3% BCR-ABL1-positive cells in blood samples from healthy donors in a dilution series. While the PLA-flow could clearly detect positive cells at a dilution of 1:1000, some BCR-ABL1-positive cells were also seen above background at a dilution of 1:10,000 (Fig. 5).

**Figure 6:**
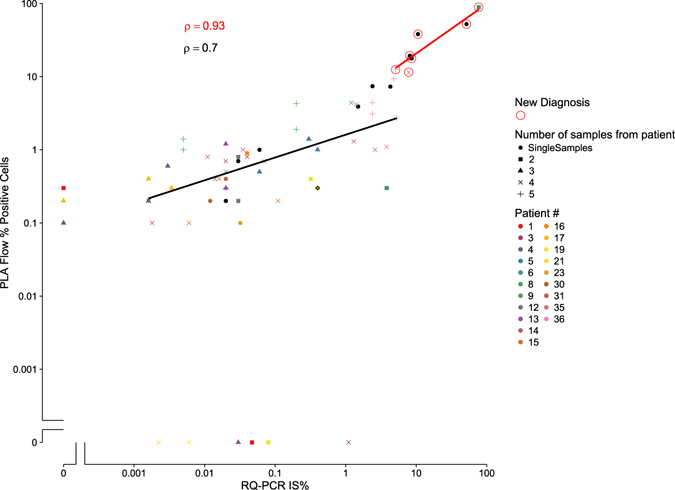
Correlation of BCR-ABL1 fusion protein positive cells as determined by PLA-flow and *BCR-ABL1* fusion transcript levels as determined by RQ-PCR. A total of 81 samples from 36 different patients were analyzed and compared; seven samples (indicated with red circles) represented samples analyzed before initiation of therapy, whereas the remaining samples were collected at follow-up. Two correlations were performed, one for follow-up samples (ρ = 0.7) and one for diagnostic samples (ρ = 0.93). Patient numbers and numbers of samples investigated for each patient is indicated with different colors/symbols.

To further compare the performance of PLA-flow with RQ-PCR, and to see how well suited PLA-flow is for MRD quantification in a routine setting, we assessed an extended series of 81 samples from 36 CML patients, all positive for the *BCR-ABL1* major variant, both newly diagnosed (n = 7) and patients that at the time of sampling were under treatment with TKIs, such as imatinib, dasatinib, nilotinib and ponatinib, or had previously undergone TKI treatment (n = 29; Supplementary Table S2). As seen in Fig. 6, a strong correlation of levels was demonstrated between the two methods; ρ = 0.93 (p = 0.0067) for samples taken at diagnosis, and ρ = 0.7 (p = <0.001) for follow-up samples. In the 51 samples with RQ-PCR values <0.1%, 43 samples showed concordant results (31 samples were positive and 12 negative with both techniques), while discordant results were observed in eight samples (five samples were positive by RQ-PCR only and three samples by PLA-flow only). Hence, PLA-flow detected most samples with MRD levels below 0.1% as determined by RQ-PCR, although discrepancies were observed. There was a strong concordance for detection via RQ-PCR versus PLA-flow among the patients. However, the levels measured by the two assays were not strictly correlated. This is not surprising since total RNA levels on the one hand, and numbers of cells positive for the fusion protein on the other, may well vary independently if numbers of transcripts per cell vary in different patients. We are currently planning single cell analyses of RNA and protein levels in patients to investigate if such variation can be demonstrated between individual patients, and perhaps as an effect of therapy.

### Combining PLA-flow and CD34 staining

**Figure 7:**
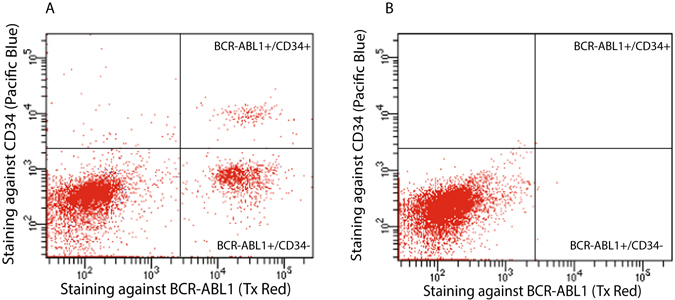
Simultaneous staining of CML cells for BCR-ABL1 and for CD34. (**A**) The image displays simultaneous immunofluorescence staining for CD34 during BCR-ABL1 PLA-flow in a newly diagnosed CML patient. Three distinct populations were observed: A major population negative for both BCR-ABL1 and CD34, one subpopulation (13.8%) expressing BCR-ABL1 but not CD34, and one subpopulation (1.8%) positive for both CD34 and BCR-ABL1. (**B**) The same experiment was performed on a healthy individual as a negative control. The X-axes represent intensity of staining against BCR-ABL1 and the Y-axes reflect the CD34 staining.

Analysis of blood cells from CML patients by PLA-flow provides an attractive opportunity to simultaneously analyze other cellular markers. To explore this we used immunofluorescence staining with antibodies directed against the stem cell and progenitor marker CD34, applied before the PLA reaction. The flow cytometric analysis revealed populations of malignant cells expressing only the BCR-ABL1 fusion protein or that were double positive for BCR-ABL1 and CD34 (Fig. 7). The results underscore the potential of this technique to further characterize malignant cells expressing fusion proteins.

## Discussion

CML is currently diagnosed and monitored over time using RQ-PCR in routine practice. This standardized assay provides quantitative measurement of number of transcripts of the *BCR-ABL1* fusion gene in PB cells. The assay is known to reflect the level of residual tumor cells in patients undergoing TKI treatment^8^. Here, we present a novel approach for directly detecting and counting individual cells that carry the BCR-ABL1 fusion protein through a procedure we call PLA-flow. Similar to RQ-PCR, the PLA-flow method demonstrated a high sensitivity and specificity, hence highlighting its potential suitability for routine diagnostic testing and MRD measurements.

In many hematological malignancies, flow cytometry is the method of choice for diagnosis and monitoring the course of disease. However, to date, no method has been available to identify and quantify BCR-ABL1-positive cells using flow cytometry, at least to our knowledge. The present approach combines the advantage of flow cytometry with specific and sensitive detection of the BCR-ABL1 positive leukemic cells using the *in situ* PLA. The assay provides rapid detection of BCR-ABL1 using flow cytometry, particularly attractive in the diagnostic setting, and it represents a novel tool for identification and enumeration of BCR-ABL1-positive cells that can easily be incorporated in the current diagnostic set-up for CML and other types of leukemia along with routine morphologic and phenotypic assessment.

Taking a step further, we investigated if the PLA-flow assay could be combined with detection of other surface markers, as this would enable characterization of subpopulations among cells expressing the BCR-ABL1 protein in a patient sample. In fact, by parallel immunostaining for the stem-cell marker CD34, we detected distinct subpopulations among cells expressing the BCR-ABL1 fusion protein, according to whether or not they also expressed the cell surface marker CD34. This combined approach can thus offer novel insights into cellular dynamics involved in disease progression in patients, and in their responses to TKI treatment. This was illustrated in a recent study demonstrating that flow cytometry after immune-staining of membrane markers for aberrant cells served to predict early blast transformation in CML^20^.

For monitoring therapy response in CML, it is of outmost importance to be able to quantify also very low levels of BCR-ABL1 positive cells, also to get an early indication of increasing BCR-ABL1 levels and an impending relapse. We followed two patients over nine months from diagnosis, with similar results over time for PLA-flow and RQ-PCR. In a broader investigation, we directly compared PLA-flow and the routinely used RQ-PCR in 81 diagnostic and follow-up samples, again demonstrating a high concordance between the two methods. Considering the 51 samples that had RQ-PCR values below 0.1%, representing the clinically relevant cut-off level for a major molecular response (MMR) in CML, PLA-flow showed concordant results to RQ-PCR in the majority of samples (86%). However, in five samples only RQ-PCR was able to detect positive cells, while in another three samples only PLA-flow gave positive results. It is quite possible that the percentages reported for RQ-PCR cannot be directly translated to tumor cell percentages, since numbers of fusion transcript and fusion protein per cell may differ within and between different patients with CML. It remains to be determined if and how the prognostic value of the two assays may differ, but the ability to simultaneously assess other markers present on the leukemic cells can present a fuller picture of the patients’ malignancy.

In conclusion, we present the PLA-flow method, serving to detect and enumerate cells expressing the BCR-ABL1 fusion protein via flow cytometry, also enabling simultaneous immunostaining of other markers. Considerably larger patient series, including serial samples, will need to be analyzed to evaluate the relative merits of the PLA-flow method versus the established RQ-PCR technique, and to determine the MMR cut-off for PLA-flow. Nonetheless, this new tool holds significant potential for routine diagnostics, eventually as a stand-alone test without the need for cytogenetics, FISH or RQ-PCR. This novel technique also opens up the opportunity to study specific cell populations involved in disease progression in CML.

## Methods

### Patients and healthy controls

Peripheral blood (PB) samples were obtained from 36 CML patients. Seven samples were collected at diagnosis prior to any drug treatment, while 74 follow-up samples were collected during treatment or after treatment was terminated (Supplementary Table S2). PB samples were also obtained from age-matched healthy individuals. Bone marrow samples were obtained from one patient at diagnosis and one at follow-up during treatment. The study was approved by the Regional Ethics Committee in Uppsala (Dnr. 2010/198 U-CAN), and was conducted in accordance with the Declaration of Helsinki. Informed consent was obtained from all subjects.

### Preparation of samples from patients and healthy controls

Freshly collected whole blood was mixed with 1–2x the blood volume of 1x BD-Pharm Lyse (BD Biosciences, San Jose, USA) to lyse red blood cells. The samples were vortexed and incubated for 5 min at room temperature (RT), followed by a 3 min centrifugation at 1,200 r.p.m, after which the cell pellets were mixed with 1.5x the starting blood volume of 1x BD-Pharm Lyse, vortexed and centrifuged again for 3 min at 1,200 r.p.m. Bone marrow samples were treated as the peripheral blood (PB) samples. The cells were washed in 1x PBS, and after another centrifugation the cells were fixated and permeabilized as described below.

PB from a newly diagnosed CML patient was spiked into PB from healthy individuals. The PB samples were first mixed in a serial dilution, and then the red blood cells were lysed, followed by fixation and permeabilization and then *in situ* PLA.

### Cell culture, fixation and permeabilization

K562 and U937 cell lines, positive and negative for the BCR-ABL1 fusion protein, respectively, and commercially available from ATCC, were used to establish parameters for the assay. The cells were tested for mycoplasma using Mycoplasma Detection Kit-Quick Test (Biotool). Both cell lines, negative for mycoplasma contamination, were grown in RPMI 1640 medium supplemented with 10% fetal bovine serum (FBS), 2 mM L-glutamine and 100 U/ml-100 µg/ml penicillin-streptomycin (Sigma-Aldrich, St. Louis, USA). Cells were collected and fixated by adding formaldehyde (Sigma-Aldrich) directly to the medium to a final concentration of 1% and incubated for 10 min at RT. The cells were pelleted and vigorously vortexed in 2 ml ice cold MeOH (Sigma-Aldrich) and incubated for 10 min at 4 °C, followed by 2 washes with 1x PBS + 1% BSA (New England Biolabs, Boston, USA), before continuing with the PLA-flow protocol. During the development of the final protocol for PLA-flow several conditions of fixation and permeabilization methods were tested. The final and optimized protocol described here is suitable for both cell lines and patient material.

### Antibodies for detection of the BCR-ABL1 fusion protein

To demonstrate the fusion proteins, we used a polyclonal sheep IgG-anti-human BCR antibody, directed against Lys174-Asp331 at the N-terminus of the BCR protein, and a polyclonal goat IgG-anti-human ABL antibody directed against Ala941-Val1140 at the C-terminus of the ABL protein. These two target domains are expected to be present in all variants of the fusion protein. The antibodies were both purchased from R&D Systems, Minneapolis, USA.

### Simultaneous staining for CD34 during PLA-flow

A Pacific Blue anti-human CD34 antibody (cat. No. 343512) was purchased from Biolegend, San Diego, USA. Antibodies against the cell surface protein CD34 were added to the blood samples after fixation and a brief wash with 1x PBS. 2 µl of the CD34 antibody, starting concentration 0.1 mg/ml, was added directly to the cell pellet. After 30 min incubation at RT in the dark, 1% formaldehyde was added for another fixation for 10 min at RT. The cells were then washed, followed by permeabilization and application of the PLA-flow protocol.

### Flow cytometry readout of PLA-based detection reactions

Cells were washed by centrifugation, unless stated otherwise. Centrifugation was carried out at 1,200 r.p.m for 3 min. All PLA-flow reaction steps were performed in a volume of 100 µl. Antibodies directed against BCR and ABL1 were conjugated to oligonucleotides using Duolink *In Situ* Probemaker (Sigma-Aldrich), according to the manufacturer’s instructions. A minimum of 5 × 10^6^ cells was used for each PLA-flow reaction. Blocking was performed in 500 µl blocking buffer (Odyssey Blocking buffer, LI-COR-TBS, Lincoln, USA) for 45 min at 37 °C. After a brief centrifugation the blocking agent was decanted and PLA probes, diluted 1:50 in Duolink *In Situ* antibody dilution mix (Sigma-Aldrich), were added to the cells for a 90 min incubation at 37 °C, or overnight at 4 °C. Cells were then washed in 1x Tris-buffered saline (TBS) with 0.05% Tween20 (Sigma-Aldrich) (TBST). Hybridization of the circularization oligonucleotides and ligation were carried out by incubating the cells with 125 nM circularization oligonucleotides in ligation buffer (10 mM Tris acetate, 10 mM magnesium acetate, 50 mM potassium acetate, 12.5 mM NaCl, pH 7.5), 0.125 µg/µl BSA (New England Biolabs), 0.025% Tween 20, 0.5 mM ATP (Fermentas, Waltman, USA), 0.02 U/µl T4 DNA ligase (Fermentas) for 30 min at 37 °C. After one wash with TBST, the cells were incubated with the reagents for RCA. 0.5 U/µl phi-29 DNA polymerase (Fermentas) was mixed with RCA buffer (2x phi-29 DNA polymerase-buffer, pH 7.5 (Fermentas), 0.25 µg/µl BSA, 0.5 mM dNTP (Thermo Fisher Scientific)) and incubated for 90 min at 37 °C. Cells were washed once with TBST and incubated for 15 min at 37 °C with 10 nM BodipyTR-fluorophore-coupled oligonucleotides in hybridization buffer (20 mM Tris-hydrochloride, 20 mM EDTA, 1% Tween 20, 1 M NaCl). After one wash with TBST the cells were placed in PBS and analyzed with a BD LSRII or BD Fortessa flow cytometer. The gating of the samples was first performed by placing a gate around the white blood cells using FSC and SSC. Thereafter, gates were placed around the positive events, representing cells positive for BCR-ABL1, and counted. Data analysis was performed using BD FACSDiva software version 8.0 (BD Biosciences). Spearman’s rank correlation coefficient was applied to test correlation. All relevant data are available from the authors. The RQ-PCR method in routine analysis were performed as previously described by van Dongen *et al*.^8^.

## Electronic supplementary material

Supplementary info
