# Supplementary material for: Flow Cytometric Measurement of Blood Cells with BCR-ABL1 Fusion Protein in Chronic Myeloid Leukemia
Source: Sci Rep. 2017 Apr 4;7:623. doi: 10.1038/s41598-017-00755-y (PMC5429594; doi:10.1038/s41598-017-00755-y)
Supplement: Supplementary file 1 — Supplementary info [file 41598_2017_755_MOESM1_ESM.pdf]

Flow Cytometric Measurement of Blood Cells with BCR-ABL1 Fusion Protein in Chronic Myeloid Leukemia

Liza Löf[1], Linda Arngården[1], Ulla Olsson-Strömberg[2], Benjamin Siart[3], Mattias Jansson[1], Joakim S. Dahlin[4], Ingrid Thörn[1], Lisa Christiansson[1], Monica Hermansson[1], Anders Larsson[5], Erik Ahlstrand[6], Göran Wålinder[6], Ola Söderberg[7], Richard Rosenquist[1], Ulf Landegren[1] and Masood Kamali-Moghaddam[1*]

[1]Dept. of Immunology, Genetics & Pathology, Science for Life Laboratory, Uppsala University, Sweden; [2]Dept. of Medical Science and Division of Hematology, University Hospital, Uppsala, Sweden; [3]Department of Anthropology, University of Vienna, Vienna, Austria; [4]Dept. of Medicine, Karolinska Institutet, Karolinska University Hospital, Stockholm, Sweden. [5]Dept. of Medical Science and Division of Biochemical Structure and Function, University Hospital, Uppsala, Sweden; [6]Dept of Medicine, Division of Hematology, Örebro University Hospital, Örebro, Sweden; Faculty of Medicine and Health, Örebro University, Örebro, Sweden; [7]Dept. of Pharmaceutical Biosciences, Uppsala University, Sweden.

*To whom correspondence should be addressed: masood.kamali@igp.uu.se, Uppsala University, Box 815, 752 37 Uppsala, Sweden. Phone: +46-18-4714454.

Running title: Flow Cytometric Detection of BCR-ABL1 Fusion Protein.

Supplemental Table 1. PLA-flow analysis of paired peripheral blood and bone marrow samples.

Patient #	RQ-PCR value (IS%)	PLA-flow value (blood) (% positive cells)	PLA-flow value (BM) (% positive cells)
**26 (treated with TKI)**	2.9	1.9	1.7
**32 (newly diagnosed)**	10.6	38.1	24.8

Supplemental Table 2. Clinical status of patients.

Patient #/sample #	Time since diagnosis (months)	Treatment	RQ-PCR value (IS%)	PLA-flow value (blood) (% positive cells)	Most recent WBC (10^9/L)
**1/1**	104	None	0.0	0.0	6.6
**1/2**	110	None	0.0	0.3	7.6
**1/3**	114	None	0.0	0.0	6.1
**1/4**	120	None	0.047	0.0	7.0
**2/1**	120	Imatinib	0.0	0.0	5.2
**3/1**	66	None	0.006	0.1	NA
**3/2**	75	None	0.07	0.7	10.8
**3/3**	78	None	0.11	0.2	8.0
**3/4**	84	Dasatinib	0.016	0.4	6.9
**4/1**	102	Ponatinib	0.032	0.1	NA
**4/2**	120	None	0.0	0.0	NA
**5/1**	138	Dasatinib	0.0	0.0	6.9
**5/2**	156	None	0.012	0.2	NA
**6/1**	114	Imatinib	0.0	0.0	NA
**6/2**	120	Imatinib	0.02	0.4	4.9
**7/1**	24	Imatinib	2.4	5.4	NA
**8/1**	0	None	7.82	11.5	NA
**8/2**	3	Imatinib	1.4	4.2	NA
**8/3**	6	Imatinib	2.6	1.0	NA
**8/4**	9	Imatinib	3.8	1.1	4.1
**9/1**	0	None	5.10	12.5	368
**9/2**	0.5	Imatinib	4.8	9.4	NA
**9/3**	3	Imatinib	2.4	4.4	NA
**9/4**	6	Imatinib	2.4	3.1	NA
**9/5**	9	Imatinib	5.3	2.7	3.8
**10/1**	120	None	1.5	3.9	6.2
**11/1**	144	None	0.0	0.0	8.7

**12/1**	51	None	1.1	0.0	9.2
**12/2**	54	None	0.02	0.7	9.5
**12/3**	57	Imatinib	0.04	0.4	NA
**12/4**	60	Imatinib	0.0016	0.2	5.6
**13/1**	114	None	0.0	0.1	NA
**13/2**	117	None	0.003	0.6	6.3
**13/3**	120	None	0.0016	0.2	NA
**14/1**	30	Dasatinib	0.006	0.5	NA
**14/2**	33	Dasatinib	0.3	1.4	4.1
**14/3**	36	Dasatinib	0.4	1.0	NA
**15/1**	0	None	77.0	85.9	NA
**15/2**	12	Imatinib	3.82	0.3	NA
**16/1**	51	Nilotinib	0.014	0.4	NA
**16/2**	54	Nilotinib	0.02	0.5	6.8
**16/3**	57	Nilotinib	1.2	4.4	NA
**16/4**	60	Nilotinib	0.02	0.5	NA
**17/1**	75	None	0.2	4.3	NA
**17/2**	78	None	0.2	1.9	6.3
**17/3**	81	Dasatinib	0.005	1.0	7.0
**17/4**	84	Dasatinib	0.02	0.3	NA
**18/1**	NA	None	0.03	0.7	NA
**19/1**	33	Dasatinib	0.03	0.8	NA
**19/2**	36	Dasatinib	0.03	0.2	NA
**20/1**	36	Imatinib	0.02	0.2	NA
**21/1**	147	None	0.03	0.0	NA
**21/2**	150	None	0.02	1.2	NA
**21/3**	156	None	0.02	0.3	NA
**22/1**	0	None	8.6	17.7	43.7
**23/1**	159	None	1.3	1.3	5.2
**23/2**	162	Imatinib	0.035	1.0	6.2
**23/3**	165	Imatinib	0.0018	0.1	4.4
**23/4**	168	Imatinib	0.011	0.8	NA
**24/1**	NA	NA	0.06	1.0	NA
**25/1**	0	None	8.10	19.3	6.8
**26/1**	3	Imatinib	2.9	1.9	3.4
**27/1**	0	None	51.7	52.2	140
**28/1**	96	Nilotinib	4.3	7.3	NA

**29/1**	NA	NA	0.0	0.0	NA
**30/1**	174	None	0.04	0.9	7.7
**30/2**	180	None	0.0	0.0	7.7
**31/1**	90	None	0.0	0.2	9.0
**31/2**	93	None	0.0016	0.4	8.2
**31/3**	96	None	0.0034	0.3	9.3
**32/1**	0	None	10.6	38.1	41
**33/1**	72	Nilotinib	0.4	0.3	7.2
**34/1**	6	Dasatinib	0.0	0.0	3.8
**34/2**	18	Dasatinib	0.02	0.0	19
**34/3**	24	Dasatinib	0.0	0.0	3.5
**35/1**	25	Dasatinib	0.4	0.3	NA
**35/2**	31	Dasatinib	0.32	0.4	NA
**35/3**	33	Dasatinib	0.00223	0.0	NA
**35/4**	36	Dasatinib	0.006	0.0	5.1
**36/1**	105	None	0.32	0.4	8.6
**36/2**	108	None	0.08	0.0	NA

NA= Not available
